# Beta-palmitate – a natural component of human milk in supplemental milk formulas

**DOI:** 10.1186/s12937-016-0145-1

**Published:** 2016-03-17

**Authors:** Zuzana Havlicekova, Milos Jesenak, Peter Banovcin, Milan Kuchta

**Affiliations:** 1 Department of Pediatrics, Comenius University in Bratislava, Jessenius Faculty of Medicine in Martin, Kollarova 2, Martin, 036 59 Slovakia; 2 BioMed Martin, Comenius University in Bratislava, Jessenius Faculty of Medicine in Martin, Mala Hora 4/A, Martin, 036 01 Slovakia; 3 Department of Pediatrics, University of P.J. Safarik, Faculty of Medicine, Children University Hospital, Trieda SNP 1, Kosice, 040 11 Slovakia

**Keywords:** β-palmitic acid, Fatty acids, Human milk, Infant nutrition, Milk formulas

## Abstract

The composition and function of human milk is unique and gives a basis for the development of modern artificial milk formulas that can provide an appropriate substitute for non-breastfed infants. Although human milk is not fully substitutable, modern milk formulas are attempting to mimic human milk and partially substitute its complex biological positive effects on infants. Besides the immunomodulatory factors from human milk, research has been focused on the composition and structure of human milk fat with a high content of β-palmitic acid (*sn-2* palmitic acid, β-palmitate). According to the available studies, increasing the content of β-palmitate added to milk formulas promotes several beneficial physiological functions. β-palmitate positively influences fatty acid metabolism, increases calcium absorption, improves bone matrix quality and the stool consistency, and has a positive effect on the development of the intestinal microbiome.

## Introduction

Human milk represents the optimal nutrition for a baby after birth and during the whole nursing period. Progressive discoveries of its particular components together with determining their physiological functions has allowed better understanding of this unique liquid. Besides nutritional functions, we can distinguish a whole spectrum of functions in human milk, including immunomodulatory and other physiological activities. Revealing the function and importance of particular components has allowed for improvement of modern supplemental milk formulas for infants who, for various reasons, cannot be breastfed. Modern preparations of supplemental milk nutrition try to copy and imitate the components of human milk to achieve not only its nutritional properties, but also other physiological functions that are provided by human milk to a breastfed child [1].

## Review

### Physiology of milk fat

The energy requirements of a baby are high. An important component of human milk, as in milk formulas, is fat, which presents an important source of energy (approximately 50 % of the energetic content of human milk and formulas). Up to 98 % of human milk lipids is in the form of triacylglycerols, in which saturated and non-saturated fatty acids are bound to the skeleton of glycerol. Palmitic acid, the major saturated fatty acid in human milk, usually represents about 20–25 % of human milk fatty acids. Sixty percent (according to some authors, up to about 86 %) of palmitic acid is esterified to the *sn-2* position in the triacylglycerols (the so-called β-position) [2–4]. The shortened name for palmitic acid bound to glycerol in the β-position (*sn-2*) is β-palmitate. However, in the majority of supplemental milk formulas, with vegetable oils as commonly used source of fat, have palmitic acid bound to the 1^st^ or 3^rd^ carbon of glycerol (*sn*-1 and −3 positions). [4]. Cow’s milk, as well as plant fats, have lower content of β-palmitate compared to human milk (cow’s milk only about 40 %, plant oils even only 5–20 %) [5]. Besides saturated fatty acids, milk also contains polyunsaturated fatty acids with a long chain, as well as essential fatty acids (linoleic acid and α-linoleic). The balanced content of these fatty acids is necessary for the correct maturation of the nervous system and for visual perception, as well as formation of important biologic mediators, e.g. eicosanoids [6, 7].

The importance of the *sn-2* bond is in the regulation of digestion and subsequent fat absorption. The first enzyme that digests fats is gastric lipase, which is already well developed in newborns. Subsequently, pancreatic lipase continues fat digestion in the gut, but a certain immaturity of the exocrine function of the pancreas is observed in neonates. Bile-salt stimulated lipase from human milk has an important role in breastfed infants. Gastric lipase accounts for approximately 10 % and lipase stimulated by biliary salts accounts for 20–40 % of fat digestion [8]. Pancreatic lipase separates fatty acids in the *sn*-1 and *sn*-3 positions, while the middle position is relatively resistant to lytic activity of this enzyme [9]. When the activity of pancreatic lipase in the intestine is appropriate, the final result of digestion of fats is free fatty acids and 2-monoacylglycerol, which subsequently creates micelles with biliary acids and is absorbed quickly. However, free saturated fatty acids with a long chain (e.g. palmitic acid), and a sufficient amount of calcium in the intestinal lumen, create non-soluble calcium soaps and so lower the overall availability of calcium for the child. If palmitic acid is bound in the *sn-2* position to glycerol, it does not create compounds with calcium, but is absorbed [5, 10]. If infants are fed with fats containing mainly palmitic acid located in the sn-1 and sn-3 position, the insufficient pancreatic lipase activity increases the risk of forming poorly absorbed calcium soaps.

This is the reason why, according to the pattern of human milk, a high content of palmitic acid in the *sn-2* position in milk formula leads to higher absorption and efficiency of palmitic acid in comparison with supplemental milk formulas with triacylglycerols derived from vegetable oils that are predominantly in the *sn*-1 and *sn*-3 position [11]. Several studies have revealed that fat from human milk is better absorbed than fat from supplemental milk formulas, while increasing the β-palmitate in formulas aims to achieve the level of fat absorption from formulas at that from human milk. The content of fatty acids bound in the β-position to glycerol is distinctly different in various supplemental milk formulas [3]. Furthermore, when analysing various supplementary milk formulas, there were significant differences in the stereospecific structure of fatty acids, as well as in the profile of particular fatty acids in comparison with human milk [4]. Regarding palmitic acid, although differences were not that great in its content, there were differences in the proportion of its binding in the *sn-2* position. Similarly, the content of fatty acids in human milk changes depending on the mother’s diet [12]. The gradual identification of particular components of human milk, as well as determination of their physiological importance, has led to adjustments of supplemental milk formulas with the aim of providing for infants who cannot be breastfed, for various reasons, similar benefits as those of human milk. Besides adjustment of the content of particular immunomodulatory (nucleotides, prebiotics, oligosaccharides, probiotics, vitamins) and other essential components with metabolic and other physiological functions (polyunsaturated fatty acids, carnitine, choline, taurine, minerals, vitamins) attention is increasingly turning towards the fatty components of cow’s milk [1]_._

Under physiological conditions, palmitic acid is an important component of human milk. On the basis of available analyses, an important part of palmitic acid is bound to the skeleton of glycerol in triacylglycerols in the *sn-2* position (position β), which has important physiological and metabolic implications in breastfed infants in comparison with infants fed supplemental milk formula [13].

In addition, changes in the position of palmitate on the molecule of glycerol may influence the presentation of fats in the plasma and their metabolism [14, 15]. However, some authors have suggested that differences in the physical characteristics of fats resulting from inter-esterification and changes in triacylglycerol structures are key determinants of the level of postprandial lipaemia, rather than the position of the fatty acid in triacylglycerols [16].

### Positive effects of β-palmitate in infants

The influence of the binding of palmitic acid at various positions to a molecule of glycerol in fats has been investigated in animals and also in human studies, with infants born prematurely or at term. The studies investigated the influence of a higher content of β-palmitate on calcium metabolism, digestion and absorption of fats, creation of bone matrix, consistency of stools and other parameters. Regarding the availability of synthetic β-palmitate, as well as formulas with its increased content, the evidence for a positive effect of this unique, from human milk-derived fat is increasing (Table 1).

**Table 1:** Characteristics of studies investigating the influence of β-palmitate on infant’s health

Authors [Reference]	Patients	Duration of the study	Type of study	Results
Bongers et al., 2007 [25]	38 constipated infants (3–20 weeks) randomised to standard formula (*n* = 18) and modified formula (*n* = 20)	3 weeks	RCT	Significant tendency towards softer stools in constipated infants fed formula containing *sn-2* palmitic acid, mixture of prebiotic oligosaccharides and partially hydrolysed whey protein; no difference in stool frequency.
Carnielli et al., 1995 [18]	Preterm infants (*n* = 12)	1 week	RCT	A formula containing triglycerides similar to human milk (26 % palmitic acid, esterified predominantly to the *sn-2* position) had significant effects on fatty acid intestinal absorption, and improved mineral balance in comparison with a conventional formula.
Carnielli et al., 1996 [11]	Healthy, term infants (*n* = 9)	At least 5 weeks	RCT	Dietary triacylglycerols containing palmitic acid predominantly at the β-position, as in human milk, had significant beneficial effects on the intestinal absorption of fat and calcium in healthy term infants.
Innis et al., 1994 [2]	Breastfed (*n* = 17) and formula-fed infants (*n* = 18)	3 months	Not specified	16:0 triacylglycerols were absorbed from human milk similar to *sn-2* monoacylglycerols.
Kennedy et al., 1999 [20]	Healthy, term neonates fed with standard formula (*n* = 103), high *sn-2* formula (*n* = 100) and breastfed infants (*n* = 120)	12 weeks	RCT	Infants receiving high *sn-2* formula, similar to breastfed infants, had higher bone mineral content, softer stools and a lower proportion of stool soap fatty acids than infants receiving a standard formula.
Litmanowitz et al., 2013 [21]	83 term infants fed with high β-palmitate formula (*n* = 30), standard formula (*n* = 28) and breastfed (*n* = 25)	12 weeks	RCT	Infants consuming high β-palmitate formula had changes in the bone speed of sound that were comparable to breastfed infants and favourable compared to infants on low β-palmitate formula.
Litmanowitz et al., 2014 [30]	Formula-fed infants with high β-palmitate (*n* = 21), standard vegetable oil mix (n = 21) or breastfed infants (*n* = 21)	12 weeks	RCT	Consumption of a high β-palmitate formula, comparable to breast-feeding, affects infant crying patterns during the first weeks of life.
Lopéz-Lopéz et al., 2001 [3]	36 healthy full-term infants: 12 infants fed with human milk, 12 infants fed with formula containing 19 % palmitic acid esterified in the β-position (α-formula) for 2 months, 12 infants fed with the α-formula during the first month and with the β-formula (44.5 % palmitic acid in β-position) during the second month	2 months	RCT of formula-fed infants, non-randomised group of breastfed infants	Consumption of a high β-palmitate formula significantly reduced the contents of total fatty acids and palmitic acid in faeces.
Lopéz A. et al., 2002 [36]	120 term infants with different formulas (40 colostrum, 40 transitional milk, 40 mature milk, 11 infant formulas)	15 days	RCT of formula-fed infants, non-randomised group of breastfed infants	Human milk in Spain had low saturated fatty acids, high monounsaturated fatty acids and high linolenic acid. Infant formulas resemble the fatty acid profile of human milk, but the distribution of fatty acids at the *sn-2* position was markedly different.
Lucas et al., 1997 [27]	24 preterm infants fed with formula that differed in content of palmitate in the *sn-2* position in the formula (74, 8.4 and 28 %).	4 days	RCT	Use of a formula rich in *sn-2* position palmitate improved palmitate absorption, reduced the formation of insoluble calcium soaps in the stool, and improved calcium absorption.
Nelson and Innis, 1999 [15]	87 healthy, full-term infants (40 breastfed, 22 fed with the standard formula and 25 fed with the formula containing synthesised triacylglycerol (39 % of the 16:0 esterified at the triacylglycerol 2 position).	120 days	RCT of formula-fed infants, non-randomised group of breastfed infants	50 % of the dietary triacylglycerol 2-position 16:0 is conserved through digestion, absorption and chylomicron triacylglycerol synthesis in breastfed and formula-fed Infants. Infants fed the synthesised triacylglycerol formula had significantly lower HDL-cholesterol and apolipoprotein A-I and higher apolipoprotein B concentrations than infants fed the standard formula.
Nowacki et al., 2014 [24]	165 healthy, term infants fed with standard formula (*n* = 54), formula containing high *sn-2* palmitate (*n* = 56), or formula containing high *sn-2* palmitate plus oligofructose (*n* = 55), and 55 breastfed infants	25–45 days	RCT of formula-fed infants, non-randomised group of breastfed infants	Increasing *sn-2* palmitate in infant formula reduced stool palmitate soaps. A combination of high *sn-2* palmitate and oligofructose reduced stool palmitate soaps, total soaps and calcium, while promoting softer stools.
Quinlan et al., 1995 [23]	20 formula-fed and 10 breastfed infants	7 days	Single blinded trial	Differences in lipids between formula- and breastfed infants’ stools were due almost entirely to FAs (mainly C16:0 and C18:0) excreted as soaps. FA soaps, predominantly saturated, accounted for one-third of the stool dry weight. These data support the hypothesis that calcium FA soaps are positively related to stool hardness.
Savino et al., 2006 [29]	199 formula-fed infants with infantile colic fed with standard formula and simethicone (*n* = 103) and special formula (partially hydrolysed whey proteins, mixture of oligosaccharides, low lactose level and modified vegetable oil with 41 % palmitic acid in the β-position and starch, *n* = 96)	14 days	RCT	The use of special formula (containing partially hydrolysed whey proteins, prebiotic oligosaccharides with a high β-palmitic acid content) reduced crying episodes in infants with colic compared with a standard formula and simethicone
Yao et al., 2014 [26]	300 healthy, formula-fed, term infants, fed with four formulas: standard formula (*n* = 75), high *sn-2* palmitate term infant formula (*n* = 74), an identical formula supplemented with oligofructose at two concentrations (3 vs. 5 g) (*n* = 76, *n* = 75), and breastfed infants (*n* = 73)	8 weeks	RCT	High *sn-2*-palmitate formulas led to reduced stool soaps, softer stools and increased *bifidobacteria*, whereas addition of oligofructose further improved stool consistency.
Yaron et al., 2013 [34]	36 term infants: 14 breastfed, 22 formula-fed who were randomly assigned into high β-palmitate (*n* = 14) or low β-palmitate (*n* = 8) formula-fed infants	6 week	RCT	High β-palmitate formula beneficially affected infant gut microbiota by increasing the *Lactobacillus* and *bifidobacteria* counts in faecal stools.

### The impact of β-palmitate on calcium metabolism

Calcium is an essential mineral, especially at the time of intensive growth and skeleton formation. The stools of infants fed supplemental milk formula with a higher content of β-palmitate have a comparable amount of calcium and also fatty acids as those of fully breastfed infants. This amount was distinctly lower, compare to the infants fed a supplemental milk formula containing little β-palmitate. From a practical point of view, it is evident that β-palmitate in the supplemental milk formula, similar to its function in human milk, positively influences calcium metabolism by increasing its absorption from the intestinal lumen and positively influencing mineralization of the growing skeleton [3]. In an animal model, a β-palmitate-rich formula increased calcium absorption through the increase of its solubility in the small intestinal content [17]. The high content of β-palmitate in supplemental milk formula lowers the amount of calcium in the stool and subsequently, there is higher excretion in the urine [18]. There is a significant direct relationship between the amount of β-palmitate in supplemental milk formula and the grade of calcium absorption, as well as a decrease in the creation of calcium soaps and the increased grade of absorption of fatty acids [13].

### The impact of β-palmitate on the bone matrix

Early childhood is critical for the optimal development of bone matrix mineralization and diet significantly influences this complex process [19]. As already mentioned, a higher content of β-palmitate has a positive impact on calcium absorption. In a study with 100 infans who were fed milk formula with 50 % proportion of β-palmitate, the whole body mass of bone mineral was compared and evaluated densitometrically against fully breastfed infants and infants fed a standard milk formula with a low content of β-palmitate. The formula with a high β-palmitate content led to a significantly higher mass of bone mineral in comparison with the group fed the control standard formula. In contrast, there was no difference between the group with β-palmitate and the fully breastfed infants [20]. A recent double-blind controlled study analysed the impact of supplemental nutrition with various contents of β-palmitate on anthropometric parameters and bone mass in a group of infants born at term. Quantitative ultrasound measurements of bone speed of sound (SOS) are an important tool for the diagnosis and follow-up of bone strength in infants. SOS is directly proportional to the quality, strength and density of the bone matrix. The results showed that breastfed infants and infants fed a formula with a high content of β-palmitate had a significantly higher bone speed of sound compared to infants fed a standard milk formula with a low content of β-palmitate [21]. However, further studies are needed to confirm this effect and to analyse the effect of β-palmitate supplementation in supplemental milk nutrition on bone matrix in the long term.

### Influence of β-palmitate on the absorption of fatty acids and stool consistency

Increased absorption of palmitate at its binding in *sn-2* position to the glycerol skeleton could present an important source of energy for a child. Increasing β-palmitate content leads to a positive influence of the resorption of fatty acids, as well as their spectrum in plasma being more similar to observations in fully breastfed infants [2, 22]. The formation of insoluble calcium soaps is responsible for a harder stool [23]. In addition, calcium in this form is unavailable for a child and it is excreted from the body in the stool. Breastfed infants have a softer stool in comparison with infants fed standard formulas with a small amount of β-palmitate [11, 20, 24]. With increasing β-palmitate content, due to better absorption of fatty acids and a higher proportion of absorbable 2-monoacylglycerols, the risk of formation of insoluble calcium soaps decreases with subsequent improvement in the stool consistency, which was softer without an increase in the overall stool volume [20]. In a small double-blind randomised cross-over trial in term infants with constipation, a new formula containing a high concentration of β-palmitate, as well as a mixture of prebiotic oligosaccharides and partially hydrolysed whey protein, resulted in a strong tendency towards a softer stool in constipated infants, but no difference in defecation frequency. Therefore, this formula may be recommended for constipated infants [25]. A limitation of this study is a lot of variables influencing stool consistence. Recent double-blind studies in healthy term infants indicate that high β-palmitate formula led to reduced stool soap formation and softer stools, and this effect was more visible with the addition of prebiotic oligofructose [24, 26].

It might be said that the high content of β-palmitate in supplemental milk formula positively influences metabolism of fatty acids and their absorption from the intestinal lumen with a subsequent energetic improvement and mineral balance. Newborns and infants in the first few weeks of life still have some immaturity of the pancreatic lipase system and therefore, adding β-palmitate to the supplemental milk formula by-passes this transient physiological insufficiency by providing sufficient absorption of fatty acids, as well as calcium. Similar results with improvements in absorption of fatty acids, lowering of calcium waste in the stool due to its increased resorption, and subsequent softening of the stool has been observed by other authors [11, 24, 27]. Several studies have shown that palmitic acid can be efficiently absorbed, thus avoiding fatty soap formation if it is present in the *sn-2* position [28]. Observations that an increased content of β-palmitate leads to lower absorption of essential polyunsaturated fatty acids have not been confirmed subsequently The formula with a higher content of β-palmitate was well tolerated and had no negative impacts on growth [20, 22].

### Impact of β-palmitate on the duration of crying and on sleep

In a randomised controlled study the use of a partially hydrolysed whey proteins formula supplemented with prebiotic oligosaccharides and with a high β-palmitic acid content induces a reduction of crying episodes in infants with colic compared with a standard formula and simethicone. Regarding the fact that the control formula, besides β-palmitate, also lacked oligosaccharides and whey hydrolysate, this observed clinical effect might be partially attributed to these other components [29]. In a double-blind randomized clinical trial a significant reduction of crying duration was observed in the group of infants fed a formula with a high content of β-palmitate in comparison with infants fed a control low-β-palmitate formula. There was no significant difference in crying pattern between breastfed infants and infants fed a formula with a high content of β-palmitate [30]. In another study with formulas supplemented with high β-palmitate content, a significantly lower number of crying infants, as well as shorter duration of crying episodes during the day and night, was observed. The reduction of crying was observed especially during the evening and night, which effect might be explained by a complex mechanism of β-palmitate activity, namely establishment of circadian biorhythms of a child and with a positive influence on the system of neuroendocrine mediators and regulators [13]. There may also have been an effect due to the softening of stools and thus a decrease in the gastrointestinal discomfort in relation to solid stool.

### The impact of β-palmitate on intestinal microflora

An important component of the intestine is the intestinal microbiome (microflora), which is an important “organ” with many functions not only at the level of the intestine, but also at the level of the whole organism, e.g. modulation of the inflammatory and immune response, prevention of colonisation by invasive pathogens, creation of essential compounds for the organism (e.g. vitamins, short-chain fatty acids), participation in the digestion of some nutrients, as well as regulation of intestinal maturation of the intestine and proliferation of intestinal epithelial cells [13]. After birth, the child has a sterile intestinal lumen, which is rapidly colonised by particular microorganisms, and the character of the intestinal microbiome may have important consequences regarding the prevention or origination of some pathological diseases, including gastrointestinal diseases, as well as extraintestinal diseases (e.g. allergic diseases, neuropsychiatric diseases and inflammatory intestinal diseases) [31–33]. An important factor modulating the creation of the intestinal microbiome is the nutrition of the child, and from this point of view, we consider human milk as an ideal creator and regulator of the physiological intestinal microbiome. In a recent clinical study, a positive influence of a high content of β-palmitate in supplemental milk formula was found as it led to an increase in *Lactobacillus* and bifidobacteria with subsequent correct intestinal maturation with antagonism of pathogenic bacteria and positive immunomodulatory effects [34]. The most recent double-blind study in 300 healthy term infants also showed that a high content of β-palmitate in formula resulted in higher faecal bifidobacteria concentration and improved stool consistency, and that there was no difference compared with human milk-fed infants [26]. These studies also showed an immunomodulatory effect of β-palmitate via a positive influence of the intestinal microbiome. Further studies are needed.

### The influence of β-palmitate on inflammatory processes in the intestine

Interesting results were shown by an experimental study using an animal model in which the possible protective effect of β-palmitate on the development of intestinal inflammation was studied. As a model, a mouse deficient in the creation of mucin-2, which presents an important physiological protective barrier to the intestinal mucosa with anti-inflammatory effects, was used. In the group of deficient mice that were fed a formula with a high content of β-palmitate, a lower grade of intestinal erosion was observed, as well as other morphological changes in comparison with the group fed a formula containing plant oil with a low content of β-palmitate. The responsible mechanism could be represented by the activation of expression of antioxidant enzymes (superoxide dismutase, glutathione peroxidase) and the stimulation of T-regulatory lymphocytes (increased expression of transcription factor Foxp3), together with an increase in gene expression for PPAR-γ (peroxisome proliferator-activated receptors) and for cytokine TGF-β, which have important functions in the homoeostasis of the intestinal mucosa and regulation of the inflammatory response to various stimuli [35]. These recent observations open new views and perspectives on the use of β-palmitate in supplemental milk formulas. The anti-inflammatory potential of β-palmitate needs further investigation in humans.

## Conclusions

**Fig. 1:**
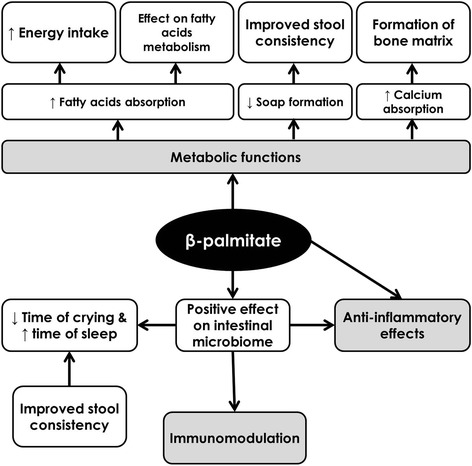
Possible beneficial effects of β-palmitic acid on children’s health

The composition and functions of maternal milk are unique and they are the basis for the development of modern supplemental milk formulas, providing the most adequate substitution for infants who cannot be, for various reasons, breastfed human milk. Besides particular immunomodulatory factors, the research is focused on the uniqueness of the fat composition of human milk with a high content of β-palmitate. On the basis of available studies, we know about several positive biologic effects of β-palmitate being added to supplemental milk formulas according to the pattern of human milk (Fig. 1). Further studies are necessary to prove these observations and, at the same time, elucidate other physiological positive effects of β-palmitate on neonate development, not only in the short term, but also in the long term.

## Abbreviations

Foxp3: Forkhead box protein P3
PPAR-γ: Peroxisome proliferator-activated receptors
RCT: Randomised controlled trial
SOS: speed of sound
TGF-β: Transforming growth factor β

## References

[1] Koletzko B, Agostini C, Bergmann R, Ritzenthaler K, Shamir R (2011). Physiological aspects of human milk lipids and implications for infant feeding: a workshop report. Acta Paediatr.

[2] Innis SM, Dyer R, Nelson CM (1994). Evidence that palmitic acid is absorbed as sn-2 monoacylglycerol from human milk by breast-fed infants. Lipids.

[3] Lopez-Lopez A, Castellote-Bargallo AI, Campoy-Folgoso C, Rivero-Urgel M, Tormo-Carnice R, Infante-Pina D, Lopez-Sabater MC (2001). The influence of dietary palmitic acid triglyceride position on the fatty acid, calcium and magnesium contents of at term new born faeces. Early Hum Dev.

[4] Straarup EM, Lauritzen L, Faerk J, Hoy CE, Michaelsen KF (2006). The stereospecific triacylglycerol structure and fatty acid profiles of human milk and infant formulas. J Pediatr Gastroenterol Nutr.

[5] Tomarelli RM, Meyer BJ, Weaber JR, Bernhart FW (1968). Effect of positional distribution on the absorption of the fatty acids of human milk and infant formulas. J Nutr.

[6] Gil A, Ramirez M, Gil M (2003). Role of long-chain polyunsaturated fatty acids in infant nutrition. Eur J Clin Nutr.

[7] Sellmayer A, Koletzko B (1999). Long-chain polyunsaturated fatty acids and eicosanoids in infants--physiological and pathophysiological aspects and open questions. Lipids.

[8] Lindquist S, Hernell O (2010). Lipid digestion and absorption in early life: an update. Curr Opin Clin Nutr Metab Care.

[9] Rogalska E, Ransac S, Verger R (1990). Stereoselectivity of lipases. II. Stereoselective hydrolysis of triglycerides by gastric and pancreatic lipases. J Biol Chem.

[10] Small DM (1991). The effects of glyceride structure on absorption and metabolism. Annu Rev Nutr.

[11] Carnielli VP, Luijendik IHT, van Goudoever JB, Sulkers EJ, Boerla AA, Degenhart HJ, Sauer PJ (1996). Structural position and amount of palmitic acid in formulas: effects on fat, fatty acid, and mineral balance. J Pediatr Gastroenterol Nutr.

[12] Jensen RG (1999). Lipids in human milk. Lipids.

[13] Bar-Yoseph F, Lifshitz Z, Cohen T (2013). Review of *sn-2* palmitate oil implications for infant health. Prostaglandins Leukot Essent Fatty Acids.

[14] Innis SM, Nelson CM (2013). Dietary triacyglycerols rich in sn-2 palmitate alter post-prandial lipoprotein and unesterified fatty acids in term infants. Prostaglandins Leukot Essent Fatty Acids.

[15] Nelson CM, Innis SM (1999). Plasma lipoprotein fatty acids are altered by the positional distribution of fatty acids in infant formula triacylglycerols and human milk. Am J Clin Nutr.

[16] Berry SE (2009). Triacylglycerol structure and interesterification of palmitic and stearic acid-rich fats: an overview and implications for cardiovascular disease. Nutr Res Rev.

[17] Lee YS, Kang EY, Park MN, Choi YY, Jeon JW, Yun SS (2008). Effectsof sn-2 palmitic acid-fortified vegetable oil and fructooligosaccharide on calcium metabolism in growing rats fed casein based diet. Nutr Res Pract.

[18] Carnielli VP, Luijendik IHT, van Goudoever JB, Silkers EJ, Boerlage AA, Degenhart HJ, Sauer PJJ (1995). Feeding premature newborn infants palimitic acid in amount and stereoisomeric position similar to that of human milk: effects on fat and mineral balance. Am J Clin Nutr.

[19] Jakusova L, Jesenak M, Schudichova J, Banovcin P (2013). Bone metabolism in cow milk allergic children. Indian Pediatr.

[20] Kennedy K, Fewtrell MS, Morley R, Abbott R, Quinlan PT, Wells JCK, Bindels JG (1997). Double-blind, randomized trial of a synthetic triacylglycerol in formula-fed term infants: effects on stool biochemistry, stool characteristics, and bone mineralization. Am J Clin Nutr.

[21] Litmanovitz I, Davidson K, Eliakim A, Regev RH, Dolfin T, Arnon S, Bar-Yoseph F, Goren F, Goren A, Lifshitz Y, Nemet D (2013). High-beta-palmitate formula and bone strength in term infants: a randomized, double-blind, controlled trial. Calcif Tissue Int.

[22] Innis SM, Dyer RA, Lien EL (1997). Formula containing randomized fats with palmitic acid (16:0) in the 2-position increases 16:0 in the 2-position of plasma and chylomicron triacylglycerols, but reduce phospholipid arachidonic and docosahexaenoic acids, and alter cholesteryl ester metabolism in formula-Fed piglets. J Nutr.

[23] Quinlan PT, Lockton S, Irwin J, Lucas AL (1995). The relationship between stool hardness and stool composition in breast- and formula-fed infants. J Pediatr Gastroenterol Nutr.

[24] Nowacki J, Lee HC, Lien R, Cheng SW, Li ST, Yao M, Northington R, Jan I, Mutungi G (2014). Stool fatty acid soaps, stool consistency and gastrointestinal tolerance in term infants fed infant formulas containing high sn-2 palmitate with or without oligofructose: a double-blind, randomized clinical trial. Nutr J.

[25] Bongers MEJ, de Lorijn F, Reitsma JB, Groeneweg M, Taminiau JAJM, Benninga MA (2007). The clinical effect of a new infant formula in term infants with constipation: a double-blind, randomized cross-over trial. Nutr J.

[26] Yao M, Lien EL, Capeding MR, Fitzgerald M, Ramanujam K, Yuhas R, Nortington R, Lebumfacil J, Wang L, DeRusso PA (2014). Effects of term infant formulas containing high sn-2 palmitate with and without oligofructose on stool composition, stool characteristics, and bifidogenicity: a randomized, double-blind, controlled trial. J Pediatr Gastroenterol Nutr.

[27] Lucas A, Quinlan P, Abrams S, Ryan S, Meah S, Lucas PJ (1997). Randomised controlled trial of a synthetic triglyceride milk formula for preterm infants. Arch Dis Child Fetal Neonatal Ed.

[28] Lien EL, Boyle FG, Yuhas R, Tomarelli RM, Quinlan P (1997). The effect of triglyceride positional distribution on fatty acid absorption in rats. J Pediatr Gastroenterol Nutr.

[29] Savino F, Palumeri E, Castagno E, Cresi F, Dalmoso P, Cacallo F, Oggero R (2006). Reduction of crying episodes owing to infantce colic: a randomized controlled study on the efficacy of a new infant formula. Eur J Clin Nutr.

[30] Litmanovitz I, Bar-Yoseph F, Lifshitz Y, Davidson K, Eliakim A, Regev RH, Nemet D (2014). Reduced crying in term infants fed high beta-palmitate formula: a double-blind randomized clinical trial. BMC Pediatr.

[31] Biedermann L, Rogler G (2009). Environmental factors and their impact on the intestinal microbiota: a role for human disease?. Dig Dis.

[32] Deveraj S, Hemarajata P, Versalovic J (2013). The human gut microbiome and body metabolism: implications for obesity and diabetes. Clin Chem.

[33] Douglas-Escobar M, Elliott E, Neu J (2013). Effect of intestinal microbialecology on the developing brain. JAMA Pediatr.

[34] Yaron S, Shachar D, Abramas L, Riskin A, Bader D, Litmanovitz I, Bar-Yoseph F, Cohen T, Levi L, Lifshitz Y, Shamir R (2013). Shaoul. Effect of high β-palmitate content in infant formula on the intestinal microbiota of term infants. J Pediatr Gastroenterol Nutr.

[35] Lu P, Bar-Yoseph F, Levi L, Lifshitz Y, Witte-Bouma J, de Bruijn ACJM, Korteland-van Male AM, van Goudoever JB, Renes IB (2013). High beta-palmitate fat controls the intestinal inflammatory response and limits intestinal damage in mucin Muc2 deficient mice. PLoS One.

[36] Lopez A, Lopez-Sabater MC, Campoy-Folgoso C, Rivero-Urgell M, Castellote-Bargallo A (2002). Fatty acid and *sn-2* fatty acid composition in human milk from Granada (Spain) and in infant formulas. Eur J Clin Nutr.

